# Participation of Multifunctional RNA in Replication, Recombination and Regulation of Endogenous Plant Pararetroviruses (EPRVs)

**DOI:** 10.3389/fpls.2021.689307

**Published:** 2021-06-21

**Authors:** Katja R. Richert-Pöggeler, Kitty Vijverberg, Osamah Alisawi, Gilbert N. Chofong, J. S. (Pat) Heslop-Harrison, Trude Schwarzacher

**Affiliations:** ^1^Julius Kühn-Institut, Federal Research Centre for Cultivated Plants, Institute for Epidemiology and Pathogen Diagnostics, Braunschweig, Germany; ^2^Naturalis Biodiversity Center, Evolutionary Ecology Group, Leiden, Netherlands; ^3^Radboud University, Institute for Water and Wetland Research (IWWR), Nijmegen, Netherlands; ^4^Department of Plant Protection, Faculty of Agriculture, University of Kufa, Najaf, Iraq; ^5^Department of Genetics and Genome Biology, University of Leicester, Leicester, United Kingdom; ^6^Key Laboratory of Plant Resources Conservation and Sustainable Utilization, Guangdong Provincial Key Laboratory of Applied Botany, South China Botanical Garden, Chinese Academy of Sciences, Guangzhou, China

**Keywords:** pararetroviruses, virus evolution, recombination, DNA repair, small RNAs, PTGS, TGS, disease resistance

## Abstract

Pararetroviruses, taxon *Caulimoviridae*, are typical of retroelements with reverse transcriptase and share a common origin with retroviruses and LTR retrotransposons, presumably dating back 1.6 billion years and illustrating the transition from an RNA to a DNA world. After transcription of the viral genome in the host nucleus, viral DNA synthesis occurs in the cytoplasm on the generated terminally redundant RNA including inter- and intra-molecule recombination steps rather than relying on nuclear DNA replication. RNA recombination events between an ancestral genomic retroelement with exogenous RNA viruses were seminal in pararetrovirus evolution resulting in horizontal transmission and episomal replication. Instead of active integration, pararetroviruses use the host DNA repair machinery to prevail in genomes of angiosperms, gymnosperms and ferns. Pararetrovirus integration – leading to Endogenous ParaRetroViruses, EPRVs – by illegitimate recombination can happen if their sequences instead of homologous host genomic sequences on the sister chromatid (during mitosis) or homologous chromosome (during meiosis) are used as template. Multiple layers of RNA interference exist regulating episomal and chromosomal forms of the pararetrovirus. Pararetroviruses have evolved suppressors against this plant defense in the arms race during co-evolution which can result in deregulation of plant genes. Small RNAs serve as signaling molecules for Transcriptional and Post-Transcriptional Gene Silencing (TGS, PTGS) pathways. Different populations of small RNAs comprising 21–24 nt and 18–30 nt in length have been reported for *Citrus, Fritillaria, Musa, Petunia, Solanum* and *Beta*. Recombination and RNA interference are driving forces for evolution and regulation of EPRVs.

## Introduction

Retroelements (class I transposable elements) can be considered to lie at the transition from an RNA to a DNA world. They are genomic DNA elements within all kingdoms, but employ reverse transcriptase for DNA synthesis using an RNA intermediate template (Xiong and Eickbush, 1990; Simon et al., 2008; Koonin et al., 2015). Besides intracellular forms, e.g., Long Terminal Repeat (LTR) retrotransposons, virion-forming elements exist that can leave the cell. These infectious retroelements developed two distinct strategies during adaptation and co-evolution with their respective hosts. Retroviruses infecting animal and human hosts encapsidate single stranded (ss) RNA, from which a linearized dsDNA molecule with LTRs is generated. Integration in the host genome by viral encoded integrase is obligatory to obtain full-length retroviral RNA (Krupovic et al., 2018). This replication scheme is also used by LTR retrotransposons, such as *Metaviridae* and *Pseudoviridae* also known as Ty3/Gypsy- and Ty1/Copia-elements, respectively. In plants, LTR retrotransposons are present. True retroviruses are lacking but plants have infective pararetroviruses (PRV), family *Caulimoviridae*. They encapsidate circular dsDNA that, after release and transport to the nucleus, forms a minichromosome allowing synthesis of terminal redundant viral RNA (Gronenborn, 1987; Hohn and Rothnie, 2013). Therefore, viral integration into the host chromosomal DNA is not required. In the cytoplasm, the viral RNA is reverse transcribed resulting in circular full-length viral DNA. Interestingly, PRV sequences can also be found inserted into plant genomic DNA and then become endogenous PRVs (EPRVs). Insertions usually comprise silenced, degenerated and/or fossil forms. Additionally, active, proliferating EPRV that can trigger virus infection exist in some hosts indicative for a recent invasion of the plant genome.

High throughput DNA and RNA sequencing, metagenomics, bioinformatics and palaeovirology have revealed the impact of viral retroelements on eukaryotic genomes and become important for understanding the origin of viruses. In cells, DNA polymerases secure the amplification of large genomes based on linear dsDNA molecules. However, relics of the “RNA world” can still be encountered such as ribosomal RNA, RNA splicing, telomerases and RNA-dependent RNA polymerases (RDR; Gilbert, 1986; Heslop-Harrison, 2000; Krupovic et al., 2018). Special attention should be paid to active, virion forming and/or infectious retroelements because of their impact on the host genomes, horizontal DNA transfer and triggering diseases. Sequence and structural analysis of the capsid proteins support the hypotheses that retroviruses, pararetroviruses and LTR retrotransposons share the same origin dating back 1.6 billion years (Krupovic and Koonin, 2017). They most likely evolved from a common ancestor that encoded the genes for a capsid protein, protease and reverse transcriptase including the RNase H domain.

Present day retroelement repeats, originating from *Caulimoviridae*, *Metaviridae*, and *Pseudoviridae* occupy distinct niches in plant genomes (Krupovic et al., 2018; International Committee on Taxonomy of Viruses Executive Committee [ICTV], 2020), and can accumulate to high numbers in genomes of angiosperms, gymnosperms and ferns (Becher et al., 2014; Geering et al., 2014; Diop et al., 2018; Gong and Han, 2018).

**FIGURE 1:**
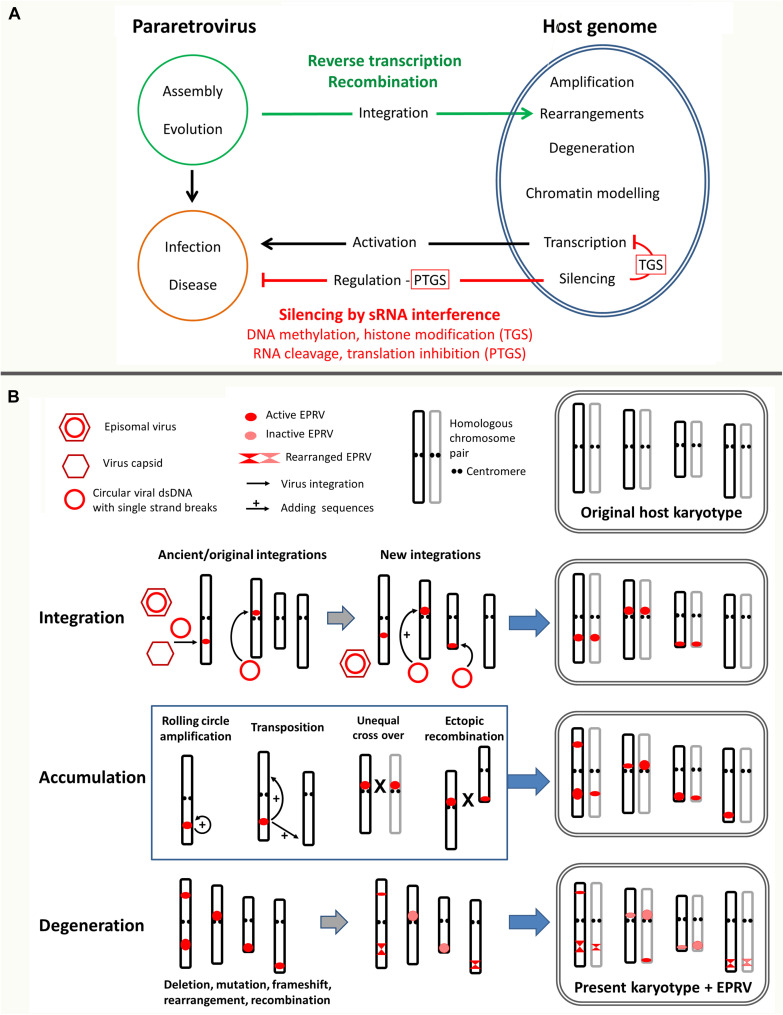
Multiple layers of endogenous pararetrovirus (EPRV) host genome interaction. **(A)** Overview of involved pathways leading to EPRV invasion of and prevalence in genomes. The ancestral pararetrovirus (PRV) most likely assembled when transcripts of a LTR retrotransposon and an infecting RNA virus recombined allowing systemic plant infection and horizontal transmission. PRVs can become residents of the host genome using illegitimate recombination or reverse transcriptase and accumulate in clusters in certain genomic regions depending on the genomic context and chromatin environment of the host. Endogenous PRVs (EPRVs) usually stay silenced and/or degenerate by mutation, rearrangement or fragmentation (see under b), but sometimes activate. When still active and complete, EPRVs can escape the cellular surveillance system by producing transcripts with terminal repeats. Those serve as template for synthesis of full length PRV episomes that can trigger a viral infection and disease, the same as original episomal PRVs. Furthermore, pararetroviral encoded suppressor of gene silencing counteract the plant defense. EPRV silencing is regulated by RNA interference comprising transcriptional and post-transcriptional gene silencing (TGS and PTGS) as well as DNA methylation and histone modifications (see Figure 2). **(B)** Detailed view of EPRV interactions with chromosomes. Prevalence of EPRVs are influenced by three steps: integration, accumulation and degeneration. Four chromosomes and their homologs in lighter shade are shown. Size of the red circle/ellipsoid indicates the number of integrations. Steps of integration, accumulation and degeneration, here depicted separately, can happen simultaneously and over a long time period. The present karyotype shows significant changes in EPRV sequences from the ancient integrations; karyotype rearrangements also occur but are not shown. **Integration:** Tandem arrays and clusters are generated by simultaneous or subsequent integration and over time through backcrosses and selfing the two homologous chromosomes homogenize. **Accumulation:** Tandemly integrated EPRVs can amplify by several mechanisms shown in the middle box: rolling cycle amplification, transposition of newly synthesized copies using reverse transcription to sites on the same or different chromosome. Unequal recombination (cross overs) between sites on homologous chromosomes or ectopic recombination between heterologous chromosomes (shown as X) will both amplify and reduce the number of copies at given site. **Degeneration:** As soon as EPRV copies are integrated, the host genome acts by inactivation through epigenetic silencing mechanisms (see Figure 2), but also through sequence degeneration by mutation and deletions that cause frameshifts, fragmentations, rearrangements and recombination.

This review points out general principles identified in EPRV evolution, adaptation, function, defense and control (Figure 1A). Special focus will be given to mechanisms related to recombination, dsDNA break repair, and RNA interference, explaining the EPRV virosphere, the space in which EPRVs occur and which is influenced by them.

## Ancient Intracellular Recombination Events

Ancient recombination events between proliferating LTR retrotransposons and co-infecting RNA viruses may have occurred in the ancestral plant cell. Indeed, analysis of encapsidated molecules of cucumber necrosis virus (*Tombusviridae*) identified also retrotransposon RNA in 0.4–1.3% of sequences isolated from virions (Ghoshal et al., 2015); such hetero-encapsidation can lead to recombination and formation of chimeric genomes. The actively transcribed *Hordeum vulgare* BARE-1 virus (*Pseudoviridae*) comprises 10% of the barley genome (Jääskeläinen et al., 2013). During its replication virus-like particles are formed that encapsidate its genomic RNA together with the reverse transcriptase, ribonuclease H and integrase (Chang et al., 2013). Since *Hordeum vulgare* BARE-1 virus lacks a movement protein for intercellular transport it stays intracellularly. Analogies regarding replication and recombination patterns of infectious retroelements have also been described for plus sense (+) ssRNA viruses (Ahlquist, 2006; Sztuba-Soliñska et al., 2011; Tromas et al., 2014).

## Leaving the Cell and Becoming a Pararetrovirus

Unequal recombination generates solo LTR footprints in the plant genome (Vitte and Panaud, 2003) that counteract bursts of retroelement replicative transposition. Such excised retrotransposon genomes might have initiated the transition to episomal replication as known from *Caulimoviridae*. An essential step toward adaptation for systemic spread within the plant host was the incorporation of genetic information for a movement protein (MP) to enable viruses to move between cells via plasmodesmata in the cell wall. Encounter of a LTR retrotransposon transcript with a + ssRNA virus encoding a 30 k MP in the cytoplasm most likely through recombination, formed chimeric molecules that gave rise to an ancestral form of *Caulimoviridae* (Koonin et al., 2015; Mushegian and Elena, 2015; Diop et al., 2018). Template switching between two RNA molecules during reverse transcription has been shown for retroviruses, LTR retrotransposons and is proposed for pararetroviruses (Froissart et al., 2005; Tromas et al., 2014; Sanchez et al., 2017). Recombination events are rare, but the rates amount to 1.4 × 10^–5^ events per site per generation for human immunodeficiency virus type 1 (Neher and Leitner, 2010) and 4 × 10^–5^ events per nucleotide site and replication cycle for the pararetrovirus cauliflower mosaic virus (Froissart et al., 2005).

The predecessors of extant members of *Caulimoviridae* might have originated from the pool of hybrid replicons. Integration loci and patterns are similar among LTR retrotransposons and EPRVs in petunia and banana (Richert-Pöggeler et al., 2003; Gayral et al., 2008). Furthermore, an integrase-like motif and quasi terminal repeats have been noticed in the *Petuvirus* petunia vein clearing virus (PVCV, Krupovic et al., 2018) that is also phylogenetically close to *Metaviridae* (Diop et al., 2018). Adaptation to episomal replication resulted in the loss of integrase function. The ancestral pararetrovirus probably gained further independence from vertical transmission by acquisition of a gene for vector transmission enabling dissemination between plants.

## Invasion of Genomes – Illegitimate Recombination

Pararetrovirus integration into plant chromosomes is supposed to occur mainly through illegitimate recombination (Liu et al., 2012; Geering et al., 2014). This can take place during somatic DNA repair or meiotic recombination. Both follow DNA double strand breaks (DSBs) that occur randomly, as a consequence of genotoxic agents or are strongly enhanced by the impact transcription has on replication fork progression (Aguilera and Gaillard, 2014; Knoll et al., 2014), or are induced deliberately during meiotic prophase (Schwarzacher, 2003). DSBs repair is vital to ensure the integrity of the genomes and is achieved by non-homologous end joining (NHJE) or homologous recombination (HR) pathways (Knoll et al., 2014; Heyer, 2015). When single DSBs are present the NHEJ is usually reliable. In case of several DSBs occurring simultaneously or if larger stretches of DNA are missing, NHEJ leads to illegitimate rejoining, often associated with deletions, random translocations, as well as insertion of sequences from elsewhere (Knoll et al., 2014; Kowalczykowski, 2015). Accordingly, pararetrovirus integration can happen if their sequences are used as template instead of homologous host genomic sequences on the sister chromatid (during mitosis) or homologous chromosome (during meiosis). Virus integration occurs frequently in somatic cells (Gayral et al., 2008), but the manifestation of such an event in reproductive cells and thus in the progeny may be rare.

In the recombination pathway, following DSBs, a single strand (ssDNA) overhang is generated by strand resection that then attracts recombinases to search for homologous sequences (Pradillo et al., 2012). This ssDNA is essential for repair and can become quite long reaching 2–10 kb when less homologous ectopic sequences are involved (Chung et al., 2010). This leads to higher fidelity in one way, as it prevents recombination within short DNA repeats next to the break, and can lead to homology recognition over a larger DNA segment that avoids recombination between homologous chromosomes in polyploids (Sepsi and Schwarzacher, 2020). On the other hand, involvement of longer ssDNA in the homology search and slower repair kinetics (Chung et al., 2010) enhance recombination with sequences located further away from the break or with external sources such as viral DNA sequences. DNA-strand invasion follows and generates a D-loop that promotes DNA strand annealing and depending on which ends are used gives rise to recombination, gene conversion or the *status quo*. Complete or partial ssDNA of pararetroviruses is present in infected plant nuclei (Hohn et al., 2008), and may indeed serve as templates for recombination or host repair machinery.

It is necessary that the DNA within the chromosome is accessible and able to unwind during repair and recombination (Stadler and Richly, 2017). Chromosome and chromatin configuration also influences where DNA breaks occur and are more frequent in particular regions (Dillon et al., 2013; Lawrence et al., 2017). ERPVs have been found accumulated in heterochromatin, in particular in AT-rich regions and next to TA dinucleotide-rich (*Oryza* sp.: Kunii et al., 2004; and Liu et al., 2012; various species: Geering et al., 2014, *Beta vulgaris*: Schmidt et al., 2021), but also next to retroelements and transposons (tomato: Staginnus et al., 2007; Petunia: Richert-Pöggeler et al., 2003; Schwarzacher et al., 2016). The latter would support an alternative mode of integration for pararetroviruses together with retrotransposons during reverse transcription when template switches can occur between viral RNA strands (Hohn, 1994).

## Prevalence of EPRV Sequences

Often EPRVs form clusters and can occupy large parts of the genome (e.g., tobacco: Lockhart et al., 2000; Petunia, Richert-Pöggeler et al., 2003; rice: Liu et al., 2012; Citrinae spp.: Yu et al., 2019; *Beta vulgaris*: Schmidt et al., 2021) and could result from the simultaneous integration of several EPRV copies in tandem or nested, or from recombination of episomal viruses with already integrated sequences (Hohn et al., 2008). Amplifications of EPRVs within the host genome can further lead to the substantial amount of EPRVs found in many plant genomes. Several mechanisms could be involved even if they occur infrequently (Figure 1B): transposition similar to retroelements (Bennetzen, 2002), rolling circle amplification (e.g., Gayral et al., 2008), as well as unequal meiotic crossing-over of tandem arrays, or ectopic recombination between EPRV clusters on non-homologous chromosomes.

In order to control EPRVs, copies are frequently inactivated by sequence degeneration or fragmentation to render transcription of entire virus components ineffective (Figure 1B). Alternatively, epigenetic silencing through methylation and small RNAs (sRNAs) has been observed (Noreen et al., 2007; Staginnus et al., 2007; Schmidt et al., 2021, and see below). Heterochromatin that is generally transcriptionally inactive and shows low recombination rates (Heslop-Harrison and Schwarzacher, 2011) therefore can be viewed as safe havens for EPRVs (Schmidt et al., 2021) and similar to retroelements can influence the genome organization and recombination landscape (Kent et al., 2017).

## RNA Interference and EPRV Control

**FIGURE 2:**
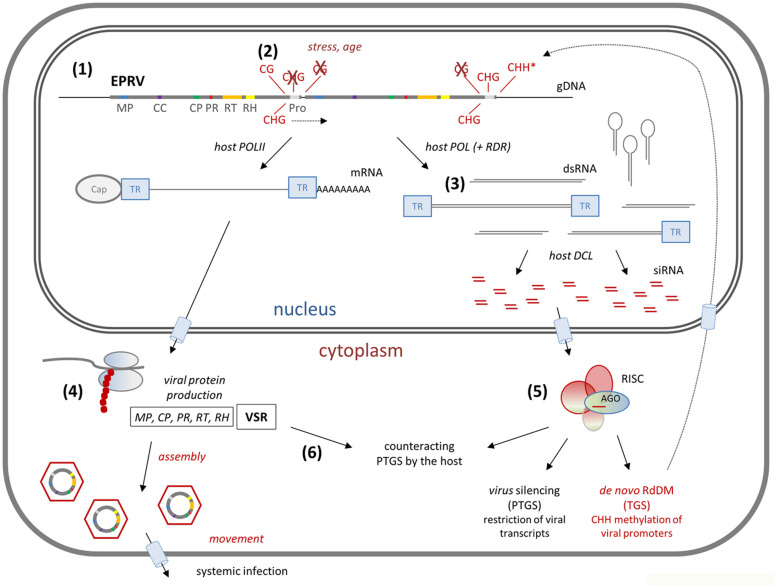
RNA interference model for inducible endogenous pararetroviruses (EPRV). (1) EPRVs in the plant genome (see Figure 1B) are usually off-frame and degraded, severely hampering full-length transcription. Sometimes, complete PRVs are integrated in tandem, as is shown here and was found for petunia vein clearing virus (PVCV) in *P. hybrida* (Richert-Pöggeler et al., 2003), through which full-length transcription can be rescued (POL = Polymerase). (2) Normally, EPRV promoters (Pro) are methylated, leading to transcriptional gene silencing (TGS); in promoters of endogenous PVCV (ePVCV) high levels of CG and CHG methylation were found (Kuriyama et al., 2020). Under the influence of stress or aging silencing can be reduced; ePVCV became transcribed and activated in older *P. hybrida*. (3) Active promoters may also lead to a diversity of incomplete viral transcripts, which serve as templates for dsRNA production, particularly via the formation of reverse complemental transcripts from reverse oriented EPRVs, secondary structures within the terminal repeats (TR), and action of host RNA dependent RNA polymerase (RDR). The dsRNAs trigger the host RNA silencing machinery, Dicer-like (*DCL)* proteins excise them into 21, 22, and 24 nt small interfering (si)RNAs. (4) Viral proteins are produced by the host ribosomal machinery, among them movement protein (MP), capsid protein (CP), protease (PR), reverse transcriptase (RT), RNase H (RH), and viral suppressors of RNA silencing (VSRs). (5) The siRNAs load onto Argonaut (AGO) proteins in RNA induced silencing complexes (RISC) to guide them to their targets: 21 nt siRNAs, produced by *DCL*4 (or *DCL*1), often load onto AGO1 to function in post-transcriptional gene silencing (PTGS) via transcript cutting; 24 nt siRNAs, produced by *DCL*3, mainly load on AGO4 to function in TGS via RNA dependent DNA methylation (RdDM) after transporting back to the nucleus. Activation of PVCV in *P. hybrida* resulted in increased levels of small RNAs and *de novo* CHH methylation (*) in the promoters of EPRVs (Kuriyama et al., 2020). (6) VSR interferes with the host silencing machinery to decrease virus degradation, ending up in an arms race between host induced silencing of the virus and viral induced silencing of the host.

Endogenous PRVs co-exist with exogenous virus(es) and viroid(s) in the same host. Like all viruses and viroid’s, EPRVs possess an RNA phase during their replication cycle (Baltimore, 1971; International Committee on Taxonomy of Viruses Executive Committee [ICTV], 2020). Activation has been reported for only a limited number of EPRVs and stresses, including from temperature, hybridization, age, or tissue culture, may activate expression of EPRVs (Ndowora et al., 1999; Lockhart et al., 2000; Richert-Pöggeler et al., 2003; Hansen et al., 2005; Kuriyama et al., 2020), as is also the case for retrotransposons (Grandbastien, 1998). Complete transcripts from genomic copies are produced if the EPRVs occur in tandem or via recombination of different segments (Richert-Pöggeler et al., 2003; Chabannes and Iskra-Caruana, 2013). (E)PRV RNA is multifunctional and serves as template for translation, for DNA-synthesis, and for RNA interference (RNAi) (Figure 2; Hohn and Rothnie, 2013; Pooggin, 2013; Prasad et al., 2019). RNAi is an evolutionary conserved, sequence-specific mechanism that regulates gene expression by employing transcriptional and post-transcriptional gene silencing (TGS and PTGS) strategies (Castel and Martienssen, 2013; Bond and Baulcombe, 2015) and is also involved in virus resistance (Wang et al., 2018). In TGS, transcription is prevented via RNA-directed DNA methylation (RdDM), while in PTGS, translation is disabled by cleavage of the transcript or translational inhibition. RNAi is a major player in antiviral defense since RNA dependent RNA polymerases (RDRs) enable a self-feeding process for systemic spreading of the RNAi signal (Obbard et al., 2009; Zhang et al., 2019; Jin et al., 2021). RNAi initiates on dsRNA molecules (Prasad et al., 2019) that are processed into small RNA (sRNA) duplexes of 21, 22 or 24 nucleotides long by plant *Dicer*(*-Like*) endoribonucleases (*DCL*s; Deleris et al., 2006). *DCL*1 functions in the production of miRNAs from imperfect double strand stems of fold-back pre-miRNAs transcribed from (endogenous) MIR-loci (Bartel, 2004; Vijverberg et al., 2016). The role of miRNAs in virus defense is thought to be either direct, by targeting the viral RNA with the possibility of 2–3 mismatches, or indirect, by triggering the biogenesis of viral (v)siRNAs (Liu et al., 2017) or regulating plant defense genes (Carbonell and Carrington, 2015). *DCL*3 and *DCL*4 produce 24-nt and 21-nt small interfering RNAs (siRNAs) from perfect dsRNA templates, respectively, each with minor distinct catalytic profiles to ensure specificity (Nagano et al., 2014). Overlapping transcripts and self-complementary regions of (E)PRVs and other DNA viruses serve as sources of dsRNA templates for vsiRNA production (Figure 2; Prasad et al., 2019). The 24-nt (v)siRNAs function in TGS via RdDM to suppress the activation of transposons and pararetroviruses (Matzke and Mosher, 2014), while the 21-nt (v)siRNAs function in PTGS via sequence-specific cleavage of transcripts (Garcia-Ruiz et al., 2010). *DCL*2 synthesizes stress-related natural-antisense-transcript (nat)-siRNAs and is thought to regulate the biogenesis of 22-nt vsiRNAs (Deleris et al., 2006). In *dcl*4 mutants, *DCL*2 is involved in increased 22-nt siRNAs production and alternate production of 22-nt siRNAs *trans-*acting (ta)-siRNA precursors (Zhang et al., 2019). The specific mode of action of 22-nt siRNAs is yet unclear, but evidence indicates that they mediate translational repression and are less effective in target cleavage (Wu et al., 2020). Recently also tRNAs have emerged as a source for small RNAs to suppress reverse transcriptase of (LTR-) retrotransposons (Schorn et al., 2017), which may imply that they act similarly against (E)PRVs. All four *DCL*s generate DNA virus-derived 21-, 22-, and 24-nt small RNAs (Blevins et al., 2006). Each of the produced sRNA classes functions by guiding an RNA-induced silencing complex (*RISC*) to its (near) complementary target sequence after being loaded into an *ARGONAUTE* (*AGO*) protein (Figure 2; Rogers and Chen, 2013). Upon arrival, other proteins from the RISC act in the degradation of the transcript, repression of its translation or in methylation to suppress its expression.

Integrated copies of PVCV are associated with repressive H3K9 methylation2 (Noreen et al., 2007) and increased CG and CHG methylation in their promoter region (Figure 2; Kuriyama et al., 2020; also found in the related *Fritillaria imperialis (Fri)*EPRV, Becher et al., 2014). Endogenous TVCV-like sequences in *Solanum* species show CHH methylation (Staginnus et al., 2007), further supporting RdDM based transcriptional silencing of EPRVs, and *Fri*EPRV showed abundant 24 nt siRNAs, a hallmark of TGS by RdRM (Becher et al., 2014). In *Beta vulgaris*, sRNAs between 18 and 30 nt were found, indicating that florendovirus beet EPRVs, although not present as active forms in nature, are nevertheless silenced by TGS and PTGS (Schmidt et al., 2021). In citrus, *Cit*EPRV was assembled from 24 nt sRNAs, while other non-endogenous viral genomes in this study were assembled from 21–22 nt sRNAs (Barrero et al., 2017). Much higher sRNA coverage of *Cit*EPRV in symptomatic plants compared to asymptomatic plants, indicates that this EPRV can also be activated and become infectious despite the host defense (Matsumura et al., 2017). Higher levels of 21 nt and 22 nt compared to 24 nt sRNAs were found for episomal badnaviruses in *Musa acuminata* (Rajeswaran et al., 2014), supporting PTGS in the presences of active viruses. Kuriyama et al. (2020) demonstrated the interaction of endogenous PVCV in *P. hybrida* with EPRV expression and host defense during development: in younger plants, petal veins are white due to the silencing of the *Chalcone Synthase* gene *CHS*-A by PTGS and the promoter sequences of integrated PVCV by TGS. In older plants TGS seems less effective permitting episomal PVCV replication transiently. Associated activity of the identified Viral Suppressor of RNA silencing (VSR) counteracted the PTGS of *CHS*-A as seen in a changed color pattern of the flowers. With more viral transcripts available also derived 21-/22-nt and 24-nt siRNAs increased, reinitiating TGS and PTGS to terminate transcription from chromosomal as well as episomal PVCV DNA (Figure 2).

## Outlook

The distribution of EPRVs observed in host genomes results from a combination of past virus integration, and the mechanisms of amplification or reduction of viral sequences integrated into the chromosomes over time (Figure 1B). EPRVs may thus be found at their initial position of invasion, or elsewhere in the genome, potentially with different (or no) site preferences.

New methodologies allow sequence analysis of ever smaller units such as virions or single cells. Thus, we can address: (i) which molecules are encapsidated?; (ii) are virions similar in their content?; (iii) what replicative and recombinogenic interactions of *Metaviridae* and EPRV occur within the cell?; (iv) which cells carry active EPRVs within tissues?; (v) what are the initial landing sites of EPRVs within chromosomes and how do they spread?; and (vi) what pathways are involved in EPRV activation and silencing?

It will be essential to characterize the presence and consequences of EPRVs across all plant species, as is happening with endogenous retroviruses in animals. Solanaceae, and in particular Petunia, with a moderate diploid genome size of 1.4 Gb comprising mobile genetic elements such as DNA transposons, LTR retrotransposons and EPRVs, easy cultivation, seed or vegetative propagation, tissue culture and transformation, along with genomic and genetic resources, is an appropriate model to help answer the questions above. Genome editing using CRISPR/Cas will be useful to elucidate the functionality of EPRVs beyond a viral context.

The diversity in EPRV structure and in co-evolution with its host requires investigation of all pools of small RNA populations present in small RNA sequencing data. Bioinformatics will be an important tool to examine the arms race and evolution of associated regulatory mechanisms in the virus and host.

## Author Contributions

KR-P in discussion with TS and KV concepted the review. KR-P wrote the abstract, introduction, outlook, and contributed to the other parts. KR-P, GC, and OA wrote the sections on PRV and EPRV evolution (1–3). TS, OA, and JH-H wrote the sections on recombination and prevalence of EPRVs in the genome (4, 5). KV wrote the section on RNAi with contributions of GC (6). TS, JH-H, and KV designed the figures. KR-P, JH-H, TS, KV, and GC edited the final manuscript. All authors contributed to writing, editing the manuscript including figures, and approved the version to be published.

## Conflict of Interest

The authors declare that the research was conducted in the absence of any commercial or financial relationships that could be construed as a potential conflict of interest.
